# Transcriptomic analysis of cumulus cells shows altered pathways in patients with minimal and mild endometriosis

**DOI:** 10.1038/s41598-022-09386-4

**Published:** 2022-04-06

**Authors:** Caroline Mantovani Da Luz, Michele Gomes Da Broi, Larissa de Oliveira Koopman, Jessica Rodrigues Plaça, Wilson Araújo da Silva-Jr, Rui Alberto Ferriani, Juliana Meola, Paula Andrea Navarro

**Affiliations:** 1 Division of Human Reproduction, Department of Gynecology and Obstetrics, Ribeirão Preto Medical School, University of São Paulo, 3900 Bandeirantes Avenue, Ribeirão Preto, São Paulo 14049-900 Brazil; 2 National Institute of Hormones and Women’s Health, CNPq, Porto Alegre, Rio Grande Do Sul 90035-003 Brazil; 3 Center for Integrative Systems Biology - CISBi, NAP/USP, Ribeirão Preto, São Paulo 14049-900 Brazil; 4 Department of Genetics, Ribeirão Preto Medical School, University of São Paulo, Ribeirão Preto, São Paulo 14049-900 Brazil

**Keywords:** Genetics, Gene expression, Gene regulation, Reproductive disorders, Infertility, Transcriptomics

## Abstract

Endometriosis is a chronic inflammatory disorder that is highly associated with infertility. This association seems to be related to oocyte impairment, mainly in the initial stages of endometriosis (minimal and mild), where no distortions or adhesions are present. Nonetheless, invasive oocyte analyses are not routinely feasible; thus, indirect assessment of oocyte quality is highly desirable, and, in this context, cumulus cells (CCs) may be more suitable targets of analysis. CCs are crucial in oocyte development and could be used as an index of oocyte quality. Therefore, this prospective case–control study aimed to shed light on the infertility mechanisms of endometriosis I/II by analyzing the CCs’ mRNA transcription profile (women with endometriosis I/II, n = 9) compared to controls (women with tubal abnormalities or male factor, n = 9). The transcriptomic analyses of CCs from patients with minimal and mild endometriosis revealed 26 differentially expressed genes compared to the controls. The enrichment analysis evidenced some altered molecular processes: Cytokine-cytokine receptor interactions, Chemokine signaling, TNF signaling, NOD-like receptor signaling, NF-kappa B signaling, and inflammatory response. With the exception of *CXCL12*, all enriched genes were downregulated in CCs from patients with endometriosis. These findings provide a significant achievement in the field of reproductive biology, directing future studies to discover biomarkers of oocyte quality in endometriosis.

## Introduction

Endometriosis is a chronic inflammatory and estrogen-dependent disorder characterized by functional endometrial-like tissue outside the uterus^1^. According to the American Society for Reproductive Medicine, endometriosis can be classified into four stages: I-minimal, II-mild, III-moderate, and IV-severe^2^. It affects 6–10% of women of reproductive age^3^, and approximately 30–50% of them are estimated to be infertile^4^. The mechanisms underlying the etiopathogenesis of infertility in patients with endometriosis remain unclear, especially in the initial stages (minimal and mild), when no distortions and adhesions in the reproductive tract are present^5^.

Several studies suggest that the infertility presented by these patients may be due to compromised oocyte quality and, consequently, embryonic quality^5–15^, or the interaction between the endometrium and the embryo^1,16^. In assisted reproduction techniques, women with endometriosis have shown decreased oocyte quality and fertilization rates^15,17^.

Nonetheless, invasive oocyte analyses are not routinely feasible since human oocytes are rarely donated to research centers, and their application in invasive techniques precludes subsequent use in assisted reproduction procedures. Thus, the indirect evaluation of oocyte quality may contribute to understanding endometriosis-related infertility^18–20^.

Indirect oocyte quality assessments are highly desirable, and, in this context, cumulus cells (CCs) may be more feasible targets of analysis. CCs are a specialized type of granulosa cells (GCs)^21^ that surround the oocyte since antrum formation^22^ and contribute to oocyte development and maturation^22,23^. CCs are closely linked to the oocyte through a network of gap junctions and paracrine signals^24,25^ and are metabolically codependent; therefore, any change in the CCs can affect oocyte quality^15,26,27^. Some studies have shown that genetic alterations in the CCs of infertile women with endometriosis may be related to lower oocyte quality^10,12,28^. However, no study has evaluated the transcriptome of CCs from infertile patients with minimal and mild endometriosis. In a previous study conducted by our group, CCs from patients with advanced (moderate and severe) endometriosis, with and without endometrioma, presented changes in the transcription profile related to acetylation, mitochondrial function (oxidative phosphorylation), and steroid biosynthesis^29^. Poli-Neto et al. (2020) demonstrated that the eutopic endometrium of women in the initial stages of endometriosis had a predominant proinflammatory profile, and the cellular microenvironments and immune cell profiles were different in the initial and advanced stages of the disorder^30^. Since the initial stages display particular morphological and physiological findings, is the transcriptome also altered as in advanced stages of endometriosis? The present study aimed to analyze the mRNA transcription profile of CCs from infertile women with minimal and mild endometriosis compared to infertile women without endometriosis to gather knowledge on the mechanisms of endometriosis I/II-related infertility.

## Materials and methods

### Ethical approval

The Research Ethics Committee of the Clinical Hospital of Ribeirão Preto Medical School approved this prospective case–control study (Protocol No. 15113/2012, approval date December 5, 2012). All methods were carried out following the Code of Ethics of the World Medical Association and the Declaration of Helsinki. All participants provided written informed consent.

### Eligibility criteria

The endometriosis group consisted of patients in the initial stages of endometriosis (minimal and mild), diagnosed by video laparoscopy, in the absence of male infertility. The control group consisted of patients who underwent diagnostic video laparoscopy to rule out the presence of endometriosis and who were diagnosed with male and/or tubal factor-related infertility.

Other eligibility criteria for both groups were age ≤ 39 years, body mass index (BMI) ≤ 34.9 kg/m^2^, and non-smoker. Participants presenting the following were excluded from the study: polycystic ovary syndrome or chronic anovulation; untreated endocrinopathies (diabetes or hypothyroidism); cardiovascular disease; dyslipidemia; rheumatologic and auto-immune diseases, and active infection.

### Controlled ovarian stimulation protocol

All patients included in this study (endometriosis I/II and control patients) underwent controlled ovarian stimulation. After synchronizing the cycles using oral contraceptive pretreatment, controlled ovarian stimulation was carried out according to the needs of each patient and following the clinical protocols adopted in our Assisted Reproduction Program, as previously described^29^.

Oocytes were retrieved from 34 to 36 h after the administration of hCG, and the luteal phase was maintained by vaginal administration of micronized progesterone (600 mg/day, Utrogestan®, Besins, Brazil).

### Sample collection

Samples were collected from all eligible patients who agreed to participate in the study from August 2014 to February 2016. The cumulus-oocyte complex was collected on oocyte retrieval, as previously described by Da Luz et al. (2021)^29^. CCs were mechanically removed by microdissection, transferred to a cryotube containing 100 μL of cryopreserves (RNAlater®, Life Technologies, USA), and stored in liquid nitrogen at -196 °C until total RNA extraction.

### Total RNA

Total RNA was extracted from the CCs using the AllPrep DNA/RNA/miRNA Kit (Qiagen®, Germany). The total RNA samples were diluted in 20 μL of the recommended solution, and the concentrations were quantified with the Qubit RNA BR Assay Kit (Invitrogen, USA) in a Qubit 2.0 Fluorometer (Invitrogen, USA). According to the manufacturer’s instructions, RNA integrity was evaluated using the Agilent RNA 6000 Nano Kit (Agilent Technologies, USA) in an Agilent 2100 Bioanalyzer (Agilent Technologies, USA). Samples with an RNA Integrity Number (RIN) ≥ 7.0 were considered appropriate.

### Pool strategy

As explained in our previous study^29^, CCs have a considerably low concentration of total RNA; thus, we chose to cluster the samples in pools, a strategy described in several reports with CCs^27,31–37^ and recommended as effective^38^. Pools of 3 patients each yielded sufficient total RNA (≥ 120 ng) and enabled to keep the pool size small, as recommended^38^. All pools had RINs ≥ 7.0.

The following clinical characteristics were considered for clustering the pools (in order of importance): age, number of oocytes collected; BMI; controlled ovarian stimulation protocol, and time after video laparoscopy. The samples in the pools were clustered heterogeneously, and the pools were homogeneous with each other. This strategy was adopted because we compared the groups, not individual samples.

### Library and RNA-Seq

Library construction was carried out following the TruSeq® RNA Sample Preparation v2 (Illumina Inc, USA) protocol. RNA-Seq was performed using the TruSeq Cluster Generation Kit v5 (Illumina Inc, USA), following the manufacturer’s instructions. The six libraries were distributed into 2 lanes and underwent paired-end sequencing (PE 2 × 101 bp) using the HISEQ2500 Illumina Platform through High Output run.

### Bioinformatics

The quality control of the nucleotide sequences was conducted using the FastQC v0.11.2 program. The PRINSEQ v0.20.4 program was used to remove nucleotides that did not meet the quality. The mapping and quantification of the reads were performed using the STAR program (v. 020,201)^39^, with GRCh37.p7 as the reference genome, and Ensembl Release 85 for gene annotation. Subsequently, the normalization and differential expression between the groups were carried out using the Bioconductor DESeq2 (version 1.15.40)^40^ in the R statistical environment^41^. We considered differentially expressed genes (DEGs) the genes with adjusted p value (FDR) < 0.05. Heatmap plots were unsupervised and generated with the aheatmap function.

### Enrichment analysis

DEGs were analyzed using the DAVID-Bioinformatics Resources 6.8. database for relevant molecular processes and pathways. The proteins encoded by the DEGs were analyzed with the STRING 11.5 database for relevant interactions and pathways. The platforms Kyoto Encyclopedia of Genes and Genomes (KEGG) and Gene Ontology (GO) were also consulted when appropriate.

### Statistics

Exploratory data analysis was performed using measures of central tendency, dispersion, and box-plot plots. Clinical characteristics (age, BMI, infertility time, and the number of oocytes collected) were compared between groups using the Mann–Whitney test. All analyses were conducted using the SAS program, version 9.4. Data were presented as median, minimum, and maximum, and significance was defined as p < 0.05.

### Ethics approval

The Research Ethics Committee of the University Hospital approved this prospective case–control study (Protocol No. 15113/2012, approval date 12/05/2012), which was carried out following The Code of Ethics of the World Medical Association.

### Consent to participate

All participants provided written informed consent (Protocol No. 15113/2012, approval date 12/05/2012).

### Consent for publication

All authors have read the manuscript and approved its publication.

## Results

### Flowchart

**Figure 1:**
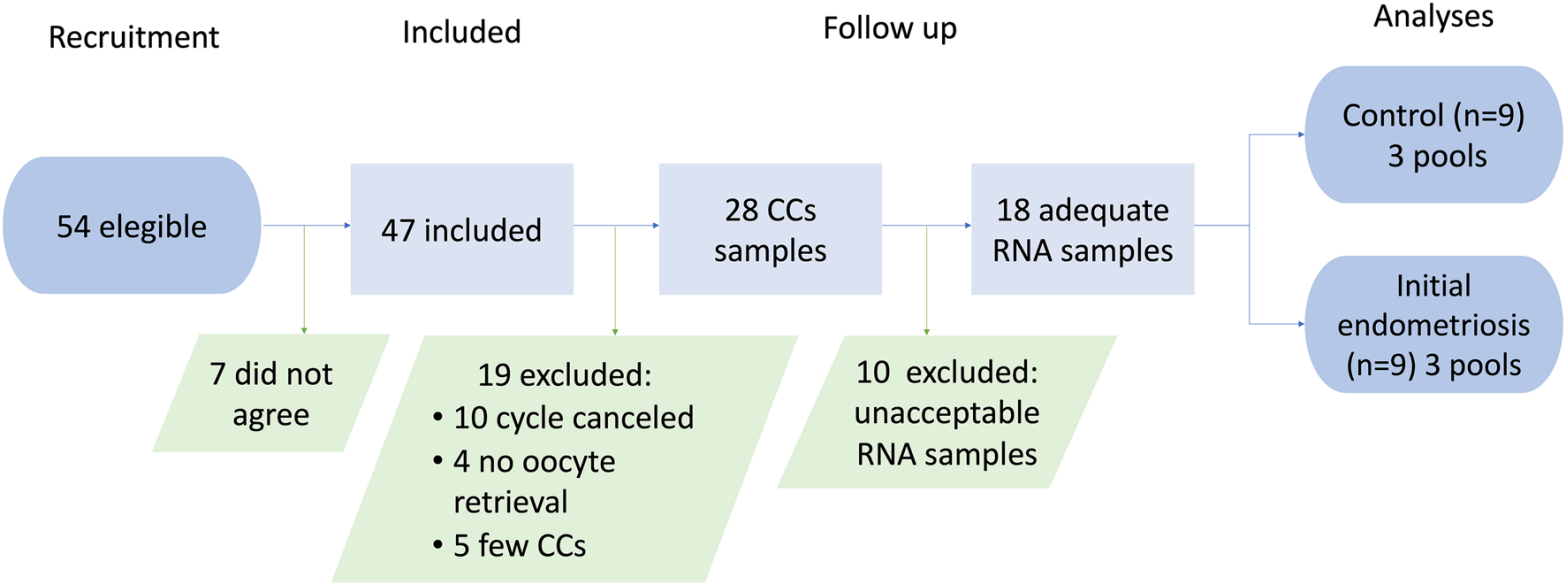
Flowchart of the study.

During the recruitment period, a total of 54 patients were deemed eligible. However, seven patients did not agree to participate in the study. Thus, 47 patients provided written informed consent and began controlled ovarian stimulation for intracytoplasmic sperm injection. Ten patients did not undergo oocyte retrieval, whereas 37 did. There was no oocyte in 4 of them, and five patients exhibited few CCs, impeding donation for the study. Therefore, the obtained CCs were donated by 28 patients. Total RNA was isolated from the CCs, and RNA integrity was assessed, although it was unacceptable in 10 samples. We obtained 18 samples, nine controls, and nine patients with endometriosis I/II; the samples were clustered in pools of three patients each. The flowchart is depicted in Fig. 1.

### Clinical variables

No significant differences between groups were observed regarding age, BMI, infertility time, and the number of oocytes (Table 1). The majority of patients (83.3%; N:15) were submitted to the flexible antagonist protocol plus rFSH or menotropin. Only 3 (16.7%; 1 control and two endometriosis I/II patients) underwent the minimal stimulation protocol.

**Table 1:** Clinical variables of women with endometriosis I/II and controls.

Clinical variables	Control (N = 9)	E I/II (N = 9)	P value
Age (years)	34 (30; 39)	36 (33; 39)	0.34
BMI (kg/m^2^)	24.23 (19.88; 30.22)	25.52 (22.60; 32.05)	0.72
Infertility time (months)	84 (24; 139)	30 (11; 192)	0.18
Number of oocytes retrieved	9 (2; 13)	8 (2; 15)	0.89

Data presented as median (minimum and maximum).

E I/II, minimal and mild endometriosis; BMI, Body Mass Index. p value < 0.05.

### RNA next-generation sequencing

**Figure 2:**
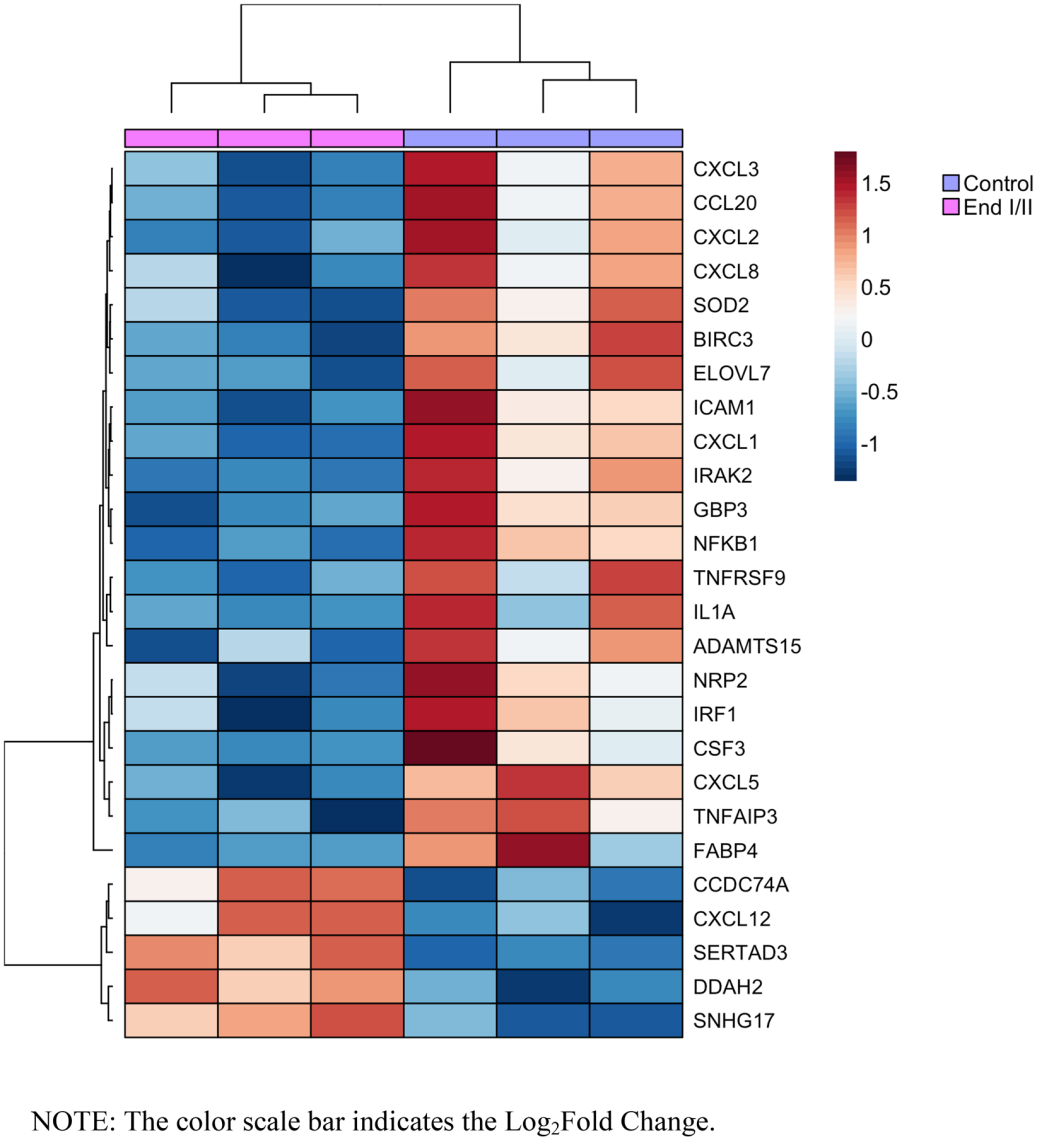
Unsupervised heatmap of the 26 DEGs of CCs from patients with endometriosis I/II compared to the controls.

Approximately 50 million reads per sample were obtained with RNA-Seq, and the average mapping quality was 92.8%. RNA-Seq provides the differential gene expression profiles in CCs of patients with endometriosis I/II compared to the controls. Such analysis enabled us to obtain a list of 26 DEGs (21 down-regulated and five up-regulated, in endometriosis I/II) with adjusted p < 0.05 as significant (Fig. 2).

### Functional enrichment analysis

The 26 DEGs of endometriosis I/II were used to perform the enrichment analysis. The main enriched pathways potentially associated with infertility-related endometriosis in the CCs were: Cytokine-cytokine receptor interactions, Chemokine signaling pathway, Tumor-Necrosis Factor (TNF) signaling pathway, Nucleotide-binding Oligomerization Domain (NOD)-like receptor signaling pathway, and Nuclear Factor (NF)-kappa B signaling pathway (Table 2). Some genes were present in more than one pathway, all of which share multiple genes. Interestingly, all enriched genes underwent a negative regulation of expression in the CCs of the patients with endometriosis I/II compared to the controls, except for the *CXCL12*, which was up-regulated. The enrichment analysis also highlighted an essential biological process, the inflammatory response with 12 altered genes (*CCL20, CXCL1, CXCL2, CXCL3, CXCL5, CXCL8, CXCL12, TNFAIP3, TNFRSF9, IL1A, IRAK2*, and *NFKB1*) (p < 0.001).

**Table 2:** In silico enrichment analysis of 26 DEGs of CCs from patients with endometriosis I/II.

Pathways	Genes	P value
Cytokine-cytokine receptor interaction	*CXCL12*^↑^*, CCL20, CXCL1, CXCL3, CXCL5, CXCL8, TNFRSF9, CSF3,* and *IL1A*	2.3e−4
Chemokine signaling	*CXCL12*^↑^*, CCL20, CXCL1, CXCL2, CXCL3, CXCL5, CXCL8,* and *NFKB1*	2.3e−7
TNF signaling	*CCL20, CXCL1, CXCL2, CXCL3, TNFAIP3, BIRC3, ICAM1* and *NFKB1*	4.5e−9
NOD-like receptor signaling	*CXCL1, CXCL2, CXCL8, TNFAIP3, BIRC3,* and *NFKB1*	3.6e−4
NF-kappa B signaling	*CXCL12*^↑^*, CXCL8, TNFAIP3, BIRC3, ICAM1,* and *NFKB1*	2.1e−6

The in silico enrichment analysis was performed using the DAVID-Bioinformatics Resources 6.8 tool and KEGG database. ^↑^ = Positively regulated. All other genes were negatively regulated. P value < 0.05.

**Figure 3:**
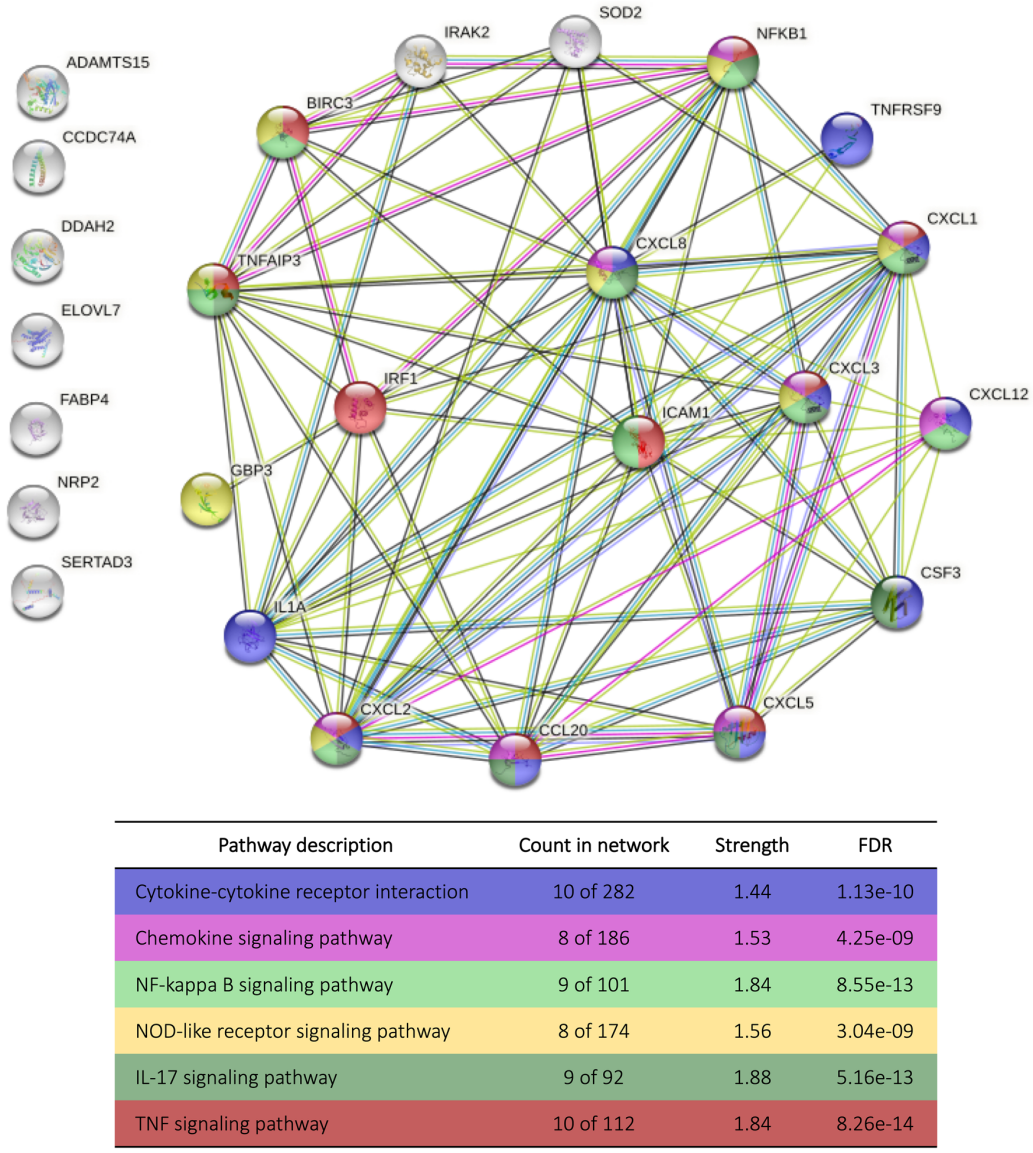
In silico enrichment analysis of 25 proteins encoded by DEGs of the CCs from patients with endometriosis I/II. *Note* Protein-coding gene = identified outside the nodes. Nodes = proteins (inside the nodes is the protein 3D structure). The colors of the nodes represent the pathways in which they were enriched. White nodes are the second shell of interactions. Edges = protein–protein associations. There are 86 edges. The white nodes on the left side, without edges, have no protein–protein associations. FDR, False Discovery Rate. Created in STRING. Permanent link: https://version-11-5.stringdb.org/cgi/network?networkId=bAvx7jSmic41.

Of the 26 DEGs, 25 were protein-coding genes. These genes encode 25 proteins, which were used to enrich protein–protein interaction networks (Fig. 3). The protein–protein interaction enrichment p value was < 1.0e−16.

The same pathways enriched using DEGs were found in the protein analysis (Fig. 3). The interaction enrichment showed that these proteins have significantly more interactions among themselves than expected for a random set of proteins of the same size and degree distribution drawn from the genome. Such enrichment indicates that the proteins are at least partially biologically connected as a group.

## Discussion

Endometriosis related to infertility seems to be associated with oocyte impairment, mainly in the initial stages of endometriosis, when no distortions or adhesions in the reproductive tract are present^6,42,43^. Exposure to hostile environments with macrophages, cytokines, and reactive oxygen species in the peritoneal and follicular fluid could lead to dysfunctional folliculogenesis and worsen oocyte quality^5,13,44,45^. Within this environment, the cross-talk between oocyte and CCs is crucial for oocyte development^24,25^. Therefore, CCs reflect oocyte status and could be used as an index of oocyte quality^10,12,27,28^. A large-scale analysis is essential to comprehend the biological changes in CCs. This study was the first to evaluate the transcriptome of CCs from infertile patients with endometriosis I/II compared to women without the disease.

The differential gene expression profile in the CCs of patients with endometriosis I/II showed 26 DEGs compared to the controls, demonstrating that endometriosis I/II is related to the deregulation of the CCs’ transcriptome. Subsequently, enrichment analysis showed altered molecular mechanisms in the CCs of patients with endometriosis I/II; Cytokine-cytokine receptor interactions, Chemokine signaling, TNF signaling, NOD-like receptor signaling, and NF-kappa B signaling. These pathways are related to immunity, and, except for *CXCL12*, all enriched genes are downregulated in endometriosis CCs. Allegra et al. (2014) also found deregulated genes in all these pathways from CCs of women with severe endometriosis by microarray analysis^27^.

It is known that endometriosis is a chronic inflammatory disease that can cause excessive reactive oxygen species (ROS) accumulation and, consequently, intra-follicular oxidative stress, even in infertile women with endometriosis I/II^46^. Lin et al. (2020) found an increase in ROS in the granulosa cells of patients with endometriosis and suggested that this process induces cell senescence, contributing to endometriosis-associated infertility^47^. This proinflammatory and ROS-filled microenvironment can trigger immune system pathways like those found in our study.

NOD-like receptors are known as recognition receptors, responsible for recognizing pathogen-associated molecular patterns released by damaged cells^48^. The activation of these receptors leads to the transcription of several genes, including *NFKB*, which induces inflammatory cytokines and chemokines^49,50^. In the ovary, cytokines and chemokines promote leukocyte recruitment and activation, steroidogenesis, follicular growth, and ovulation^51,52^. In the literature, several cytokines are found down-regulated in endometriosis CCs when compared to the controls. Moreover, follicular fluid cytokines appear to be related to successful pregnancy following IVF treatments^52^. The TNF signaling pathway acts in several processes, including cell proliferation, differentiation, and apoptosis, in addition to the modulation of immune and inflammatory responses^53^. In part of this pathway, the gene *TNFAIP6* plays an essential role in forming the extracellular matrix of the cumulus-oocyte complex^54,55^. Allegra et al*.* (2014) showed the down-regulation of the *TNFAIP6* gene in CCs of patients with endometriosis^27^. All of these pathways are essential for ovulation, as well as fertilization. The expansion of the cumulus-oocyte complex can be improved by activating Toll-like receptors, followed by genes such as *NFKB*, besides cytokines and chemokines^50^. An inflammatory process marks the rupture of the follicle. Moreover, sperm induces the release of cytokines and chemokines from CCs, enhancing the fertilization process^56,57^. Therefore, alterations in these intricate molecular mechanisms may compromise oocyte quality and decrease fertilization rates.

The main limitation of this study was its small sample size, resulting from the strict eligibility criteria adopted and the low RNA integrity and concentrations in CCs. Also, pooling samples might not be beneficial when the gene expression levels display low variability and reduce samples. However, small RNA sample pools effectively reduce the variability and compensate for the loss of replicates^38^. Furthermore, the data obtained from studies using samples collected after different controlled ovarian stimulation protocols may not necessarily be extrapolated to natural cycles.

In conclusion, the present study shows, for the first time, that endometriosis I/II could promote alterations in the transcriptome of CCs. These results provide a better understanding of the mechanisms (Cytokine-cytokine receptor interactions, Chemokine, TNF, NF-kappa B, NOD-like receptor signaling, and inflammatory response) that may affect oocyte competence acquisition in patients with endometriosis I/II. These pathways share a variety of genes and cannot be considered an individualized process. This differential transcription profile provides a significant achievement in the field of reproductive biology, directing future studies with a larger cohort to discover biomarkers of oocyte quality in endometriosis, be they pathways or genes.

## Data Availability

The RNA-sequencing data underlying this article is available in the repository Sequence Read Archive (SRA) of the National Center for Biotechnology Information (NCBI) (Permanent link: https://www.ncbi.nlm.nih.gov/sra/?term=PRJNA808988), study number: PRJNA808988. Other datasets generated during the current study are available from the corresponding author on reasonable request.
